# Factors Associated with the Practice of Low-Carb and Low-Fat Diets among Participants of the Longitudinal Study of Adult Health (ELSA-Brasil)

**DOI:** 10.3390/nu16162680

**Published:** 2024-08-13

**Authors:** Leticia Batista de Azevedo, Haysla Xavier Martins, Vivian Cristine Luft, Maria de Jesus Mendes da Fonseca, Oscar Geovanny Enriquez-Martinez, Maria del Carmen Bisi Molina

**Affiliations:** 1 Postgraduate Program in Nutrition and Health, Federal University of Espírito Santo, Vitória 29043-213, Brazil; leh.azevedo1@gmail.com (L.B.d.A.); haysla.xmartins@gmail.com (H.X.M.);; 2 Postgraduate Program in Epidemiology, Faculdade de Medicina, Federal University of Rio Grande do Sul, Porto Alegre 90035-003, Brazil; 3 National School of Public Health, Oswaldo Cruz Foundation, Rio de Janeiro 21040-900, Brazil; 4 Postgraduate Program in Nutrition and Longevity, Federal University of Alfenas, Alfenas 37130-001, Brazil

**Keywords:** public health, nutrition, dietary patterns

## Abstract

In the field of nutrition, both low-carbohydrate (LCD) and low-fat (LFD) diets were initially intended for specific subgroups but are now being embraced by the broader population for various purposes, including aesthetics and overall health. This study aims to assess sociodemographic, health, and lifestyle factors influencing diet choices among public servants in the ELSA-Brasil cohort. Diets were classified as LCD or LFD based on the Brazilian Diabetes Society (<45%) and WHO guidelines (<30%) respectively. A total of 11,294 participants were evaluated (45.3% men; 54.7% women) with a mean age of 52 ± 0.08 years. Having overweight, altered waist circumference, and a history of smoking confers higher chances of adopting an LCD compared to the usual diet, while being over 52 years, non-White race/skin color, in a lower income stratum, and having diagnosis of hypertension and/or diagnosis of diabetes mellitus decrease these chances. Regarding LFDs, belonging to the non-White race/skin color, being over 52 years old, being divorced, and practicing low physical activity decrease the chances of following such a diet compared to the usual diet. In conclusion, factors like age, socioeconomic status, health, and physical activity levels can be the key to understanding why individuals choose restrictive diets beyond clinical advice.

## 1. Introduction

Dietary patterns are extensively studied due to their associations with various health outcomes. In this context, diets stand out, as their composition can be modified to achieve specific goals, such as weight loss or the treatment and control of non-communicable chronic diseases (NCDs) [1,2].

A comprehensive analysis of disability-adjusted life years in Brazil revealed a significant shift in dietary risks. From 2010 to 2021, these risks escalated from the fourth to the second position [3]. The nation is undergoing a nutritional transition characterized by widespread consumption of ultraprocessed foods and a decline in the intake of nutritious options. These trends have a direct impact on health, contributing to clinical conditions such as overweight and obesity, both of which are significant risk factors in the development of non-communicable diseases (NCDs) [4].

Around 13.9% of the Brazilian population adheres to a restrictive diet, according to IBGE data from 2020 [5], although the motivations and influencing factors behind these dietary choices are multifaceted [6]. Furthermore, more than half of the individuals who adopt restrictive diets do so without nutritional guidance, potentially leading to undesirable health consequences [6].

Dietary patterns have been employed as tools by which to achieve various objectives, both aesthetic and health-related [7]. They can promote rapid weight loss and improve biochemical parameters [8]. Among the most common diets are those involving nutrient modification, notably, the low-carbohydrate diet (LCD) and the low-fat diet (LFD) [8,9], which have gained widespread media attention. The LFD has been studied since the 1940s, when the hypothesis emerged that saturated fats caused heart disease, leading to the promotion of an LFD for cardiovascular health. The LCD gained prominence in the 1970s with the creation of the Atkins diet and other LCD approaches, increasing media coverage and public interest in these dietary strategies [10].

The literature extensively discusses the adoption of LCDs and LFDs by the general population, emphasizing the quality of consumed carbohydrates and fats [10,11,12]. Additionally, demographic factors, such as sex, race, and age, play a role in dietary behaviors and preferences [13,14,15]. However, research specifically exploring sociodemographic, health, and lifestyle aspects related to the choice between LCD and LFD remains limited. Notably, prior studies found associations between dietary choices and habitual macronutrient intake and food preferences but no significant association linked to demographics, clinical factors, and health behaviors.

Therefore, investigating these factors has become relevant as LCD and LFD, initially intended for specific groups, are now being adopted by the general population. The objective of this article is to evaluate the sociodemographic, health, and lifestyle factors associated with these diets among public servants participating in the Brazilian cohort known as the Longitudinal Study of Adult Health (ELSA-Brasil).

## 2. Materials and Methods

### 2.1. Study Design and Population

This is a cross-sectional study conducted with baseline data (2008–2010) from the Longitudinal Study of Adult Health (ELSA-Brasil), a multicentric and prospective cohort composed of 15,105 adults. The study aims to investigate risk factors related to the incidence and progression of chronic conditions [16]. The participants include active or retired public servants, aged between 35 and 74 years, linked to public higher education and research institutions in the capitals of six Brazilian states: Salvador, Belo Horizonte, Rio de Janeiro, São Paulo, Vitória, and Porto Alegre [16].

**Figure 1:**
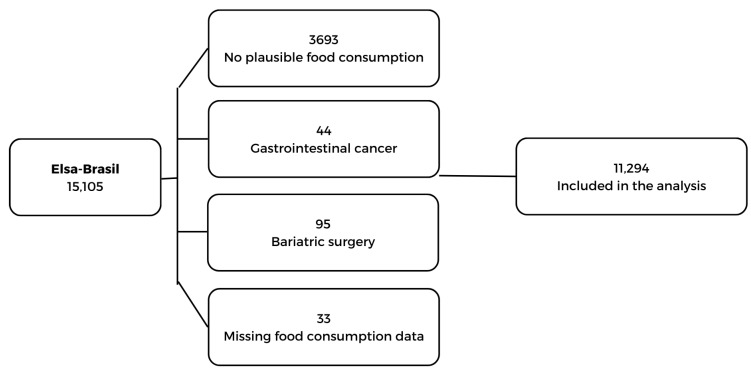
Flowchart of the study sample. Source: authors.

For the present study, individuals were excluded who did not present plausible food consumption [17], who had some type of gastrointestinal cancer (adenocarcinoma, salivary gland adenoma, mouth, colon, intestine, malt lymphoma in the stomach, prostate, pseudomyxoma peritoneal and rectum), who had undergone bariatric surgery, and for who there were missing data (referred to as losses) (*n* = 33). The final sample consisted of 11,294 participants (Figure 1).

### 2.2. Data Collection

The participants were instructed to attend the investigation center (IC) in the morning shift for clinical and biochemical examinations and interviews conducted by trained advisors. Details of the procedures are described in a previous publication [18] and below.

### 2.3. Dietary Intake

For the evaluation and quantification of dietary intake, a validated semi-quantitative Food Frequency Questionnaire (FFQ) was employed, as proposed by Molina et al. [19]. This questionnaire includes 114 food items and aims to analyze habitual consumption over the past 12 months. It is structured into four distinct sections: (1) foods/preparations; (2) portions consumed; (3) frequency of consumption, with eight response options ranging from “more than 3x/day” to “never/almost never”; and (4) reference to seasonal consumption, intended for those who spontaneously reported consuming a particular food item only during specific periods or seasons of the year [19,20].

The quantification of energy and nutrients for each food item, according to its respective reference portion, was conducted using the Nutrition Data System for Research (NDSR) software version 26, developed by the University of Minnesota (United States) [21]. The quantities of carbohydrates, lipids, monounsaturated fatty acids (MUFAs), polyunsaturated fatty acids (PUFAs), and fiber were estimated in grams based on household measures and/or the reference portion of each food item [22]. Subsequently, macronutrients were converted into percentages.

For the analysis of the present study, LCD was classified as an intake of less than 45% of carbohydrates following the Guideline of the Brazilian Diabetes Society [23], and LFD was classified as an intake of less than 30% of fat as classified by the World Health Organization [24]. Those individuals who consume above what is considered low-carb and low-fat were classified as adherents to the “usual” diet, following the classification pattern of previous studies [23,25,26].

### 2.4. Anthropometric Data

Anthropometric measurements were obtained according to standardized procedures, as established by Lohman et al. [27]. All anthropometric assessments were conducted while the participants were in a fasting state and with an empty bladder. Body weight was recorded with participants barefoot, wearing a standard uniform over their underwear, using an electronic scale (Toledo, model 2096PP, São Bernardo do Campo, São Paulo, Brazil) with a capacity of 200 kg and precision of 50 g.

Height was measured using a wall stadiometer (Seca, Hamburg, Germany) with an accuracy of 1 mm, fixed on a smooth wall without a baseboard. During the measurement, the individual remained standing barefoot, touching their head, buttocks, and heels to the wall, keeping their gauze fixed on the horizontal plane. The height was recorded during the inspiratory phase of the respiratory cycle. Based on weight and height data, body mass index (BMI) was calculated (body weight divided by height squared—kg/m^2^) [18].

Waist circumference (WC) was measured with the participant standing upright, breathing normally, feet together, upper clothing (shirt) raised, and arms crossed in front of the chest. The measurement was taken with an inextensible tape measure at the midpoint between the iliac crest and the lower edge of the last rib [18]. Elevated WC was identified when it was greater than or equal to 94 cm for men and 80 cm for women [28].

### 2.5. Sociodemographic Variables

Sociodemographic variables were obtained through a standardized questionnaire applied in face-to-face interviews. Participant age was categorized using the media as a cut-off point. Self-perception of race/skin color was recorded and characterized as “White” and “non-White” (Brown, Black, Asian, and Indigenous). Marital status was classified as “married”, “divorced”, and “single”.

The per capita family income was calculated from the total net income of the family over the last three months, divided by the number of people who depend on this income to live. Subsequently, this variable was subdivided into tertiles, representing different income strata in the sample.

### 2.6. Health and Lifestyle

During the interviews conducted at the ICs, participants self-reported information about smoking, physical activity, health status, and medication use.

Smoking status was classified into three categories: “former smoker”, “current smoker”, and “never smoked”. To measure the level of leisure-time physical activity (LTPA), the long version of the International Physical Activity Questionnaire (IPAQ), validated for Brazil, was used [29]. For the present study, the LTPA variable was recorded in minutes per week and subdivided into intensity categories: lightly active, moderate, and vigorously active.

For the classification of diabetes mellitus (DM), the presence of one of the following situations was considered: self-report of DM, use of DM medication in the last two weeks, use of insulin, glucose values (≥126 mg/dL), glycated hemoglobin (≥6.5%), and/or altered blood glucose two hours after a 75g dextrose overload (≥200 mg/dL) [23]. Regarding the classification of hypertension (SAH), the variables considered were self-report of hypertension, use of antihypertensive medications, and altered systolic (SBP) and diastolic (DBP) blood pressure (≥140 and ≥90 mmHg, respectively) [30]. Health status was classified as “good/very good”, “regular”, and “bad/very bad”.

### 2.7. Statistical Analysis

For data analysis, descriptive statistics were applied using simple frequency and percentage, as well as measures of central tendency and dispersion. Pearson’s chi-square test was used to compare proportions according to sex. Continuous variables were presented as mean ± standard deviation. The comparison of means of independent samples was conducted using the Student’s *t*-test. The binary logistic regression model was used to evaluate the association between the factors (age, race/skin color, education, income, health status, smoking, physical activity, BMI, WC, glycated hemoglobin, glucose, and DM) and outcome (LCD and LFD).

A crude model was used, adjusted for sociodemographic variables, health, and lifestyle. For the analysis of factors associated with outcomes, binary logistic regression was performed: crude model, after adjustment by model 1: sociodemographic variables (age, race/color, education, and income); model 2: model 1 + health (BMI, SAH, DM, glucose, glycated hemoglobin, WC); and model 3: model 1 + model 2 + lifestyle (LTPA, health status, and smoking). When there was collinearity, only one variable was chosen for analysis.

The level of significance for all tests was *p* < 0.05 and statistical analyses were performed using Stata 16.0 (https://www.stata.com, accessed on 2 July 2023).

## 3. Results

A total of 11,294 participants were evaluated (45.3% men and 54.7% women) with a mean age of 52 ± 0.08 years. 15.7% of women and 19.2% of men follow an LCD, while 36.8% of women and 38.0% of men have adopted an LFD. The average percentage of caloric contribution from carbohydrates in those following an LCD is 39.8% in men and 40.4% in women, compared to 54.9% and 55.7% in men and women following a usual diet, respectively. On an LFD, lipids contribute about 25.2% of the total calories for women, while for those on a usual diet, this contribution is 33.9%. In men, the lipid contribution for those on an LFD is 25.3% compared to 33.8% for those on a usual diet.

When analyzing the LCD, both sexes presented a higher proportion of individuals of White race/skin color, high income, good/very good health status, overweight, altered WC, and who reported never smoking. Additionally, in women, there was a higher prevalence of age younger than 52 years and no history of DM and SAH; while in men, a higher prevalence of low leisure-time physical activity (LTPA) was observed (Table 1).

Regarding the LFD, in both sexes, most individuals were younger, of White race/skin color, indicated good/very good health status, and had no history of SAH. Men, furthermore, predominantly had a low income, were overweight, had altered WC, and engaged in low LTPA, while most women were married, had high income, no history of DM, and reported never smoking (Table 1).

**Figure 2:**
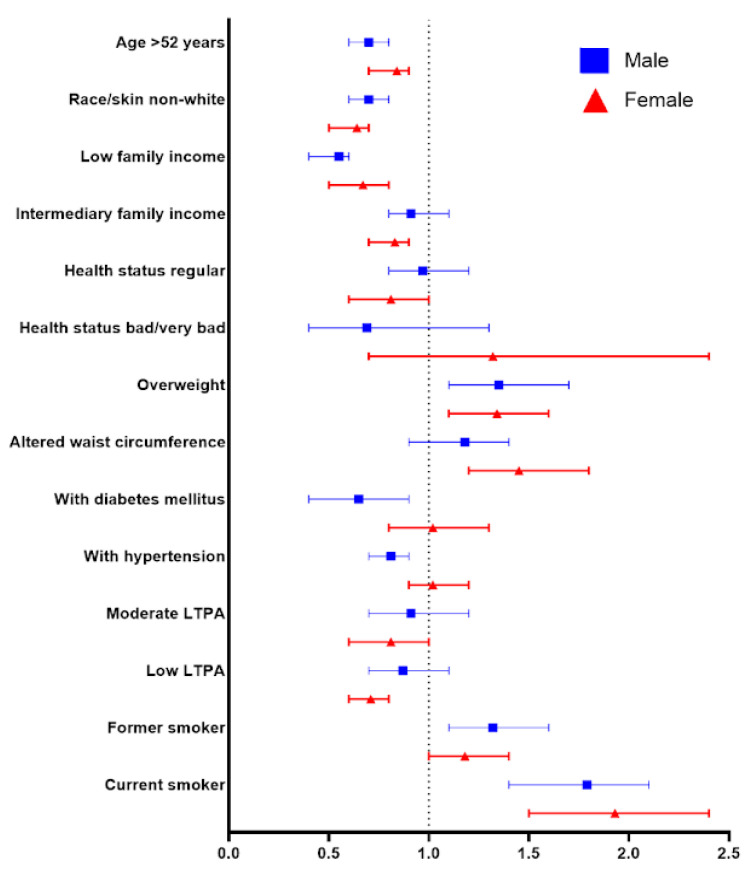
Final adjusted model with binary logistic regression for LCD.

It was observed that in both sexes, being overweight (OR = 1.35; 95% CI 1.1–1.7 [F]; OR = 1.34; 95% CI 1.1–1.6 [M]) and having a history of smoking (former smoker: OR = 1.32; 95% CI 1.1–1.6 [F]; current smoker: OR = 1.79; 95% CI 1.4–2.1 [F]; OR = 1.93; 95% CI 1.5–2.4 [M]) is associated with higher odds of following an LCD compared to the usual diet. Conversely, having low income (OR = 0.55; 95% CI 0.4–0.6 [F]; OR = 0.67; 95% CI 0.5–0.8 [M]), non-White race/skin color (OR = 0.70; 95% CI 0.6–0.8 [F]; OR = 0.64; 95% CI 0.5–0.7 [M]), and age over 52 years (OR = 0.7; 95% CI 0.6–0.8 [F]; OR = 0.84; 95% CI 0.7–0.9 [M]) decrease the chances. Additionally, in men, altered WC (OR = 1.45; 95% CI 1.2–1.8) is associated with a higher likelihood of following an LCD, while low LTPA (OR = 0.71; 95% CI 0.6–0.8) and intermediate income (OR = 0.83; 95% CI 0.7–0.9) are related to a lower probability. In women, diagnosis of DM (OR = 0.65; 95% CI 0.4–0.9) or SAH (OR = 0.81; 95% CI 0.7–0.9) are associated with a lower chance of following an LCD compared to the usual diet (Figure 2).

**Figure 3:**
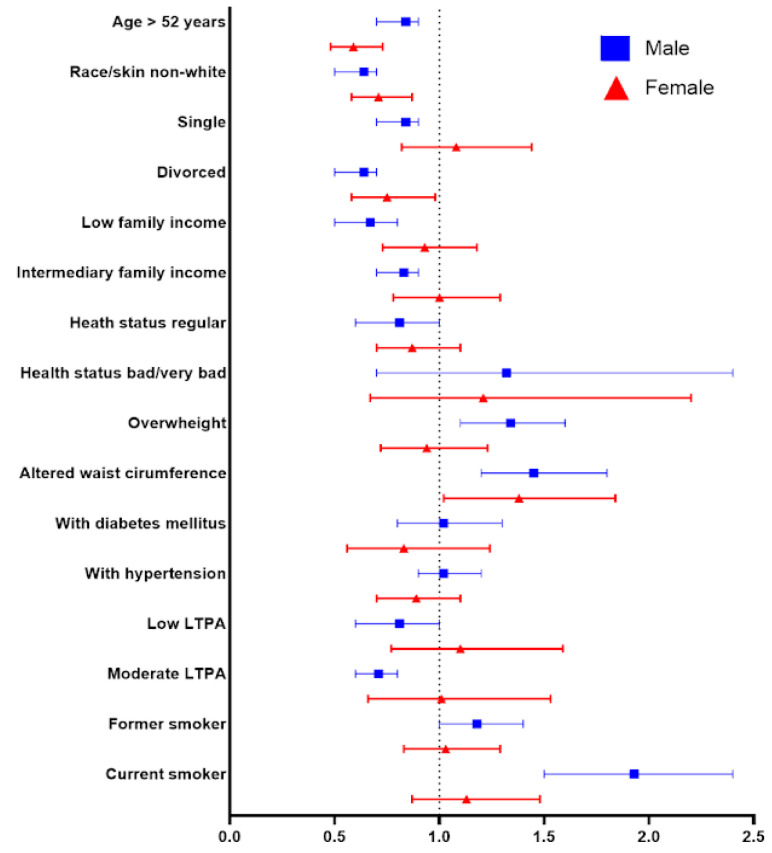
Final adjusted model with binary logistic regression for LFD.

For adopting an LFD, belonging to the non-White race/skin color (OR = 0.71; 95% CI 0.6–0.9 [F]; OR = 0.53; 95% CI 0.4–0.7 [M]) is associated with a reduction in the likelihood of following the diet in both sexes. Women over the age of 52 years (OR = 0.59; 95% CI 0.5–0.7) and those who are divorced (OR = 0.75; 95% CI 0.6–0.9) demonstrate a lower chance of adopting an LFD, while in men, these odds decrease when they engage in low LTPA (OR = 0.57; 95% CI 0.3–0.9) (Figure 3).

## 4. Discussion

The present study showed that in a sample of active and retired public servants of both sexes, having overweight status, altered WC, and a history of smoking confers higher chances of adopting an LCD compared to the usual diet, while being over 52 years, non-White race/skin color, in a lower income stratum, diagnosed with SAH and/or DM, and participating in low physical activity decrease these chances. Regarding an LFD, belonging to the non-White race/skin color, being over 52 years old, being divorced, and practicing low physical activity decrease the chances of following such a diet compared to the usual diet. The main findings stratified by sex and diet will be discussed next.

Low-carb diets

The observed association between increasing age and reduced likelihood of following an LCD can be explained by the fact that the progression of age causes cognitive decline at different levels, directly affecting appetite and taste and making dietary restrictions more challenging [31]. An association similar to our findings was observed in middle-aged and older adults in a study in the United Kingdom, this being explained by greater knowledge of food and agreement with the proposed dietary guidelines, which provide general guidelines on healthy eating habits [6].

Individuals of non-White race/color and lower income strata are less likely to adopt an LCD. Individuals with both characteristics often have more central obesity, consume lower quality diets with higher fat and simple carbohydrate intake, and engage in less physical activity, all of which contribute to metabolic implications linked to insulin resistance and an increased risk of developing type 2 diabetes mellitus [32,33]. Furthermore, socioeconomic status and race/color can influence dietary behavior due to disparities in access to health information and resources [34,35]. White individuals have greater knowledge about different nutritional strategies, as evidenced by studies in populations from the United States [36] and Europe [37], where the association between carbohydrate intake and mortality varied significantly among racial groups [38]. In a study of a nationally representative sample, past use of a low-carbohydrate diet was associated with an income higher than USD 50,000 and an age range of 50–64 [39].

A history of DM and SAH conferred a lower likelihood of following an LCD. The recommendations for adopting an LCD for the management of comorbidities are conflicting, which may lead to a preference for conventional dietary strategies [40,41]. Despite the LCD being potentially beneficial for individuals with comorbidities by assisting in glycemic control and body weight management [42], depending on the degree of carbohydrate restriction, these benefits seem to be nullified by undesirable outcomes that occur in the long run term, such as dizziness, malaise, headaches, and other conditions [6,42].

In men, low LTPA reduces the likelihood of adopting an LCD. Low-to-moderate physical activity already shows beneficial results in reducing metabolic risks and glycemic control in men, which may lead to their not undertaking restrictive diets because they understand the non-necessity of these strategies [6]. Furthermore, the LCD can impair anaerobic performance and subsequent muscle gain, which may be a concern for physically active men [43].

Being overweight and having an altered WC, exclusively in males, increase the chances of adopting an LCD, consistent with previous studies [44,45]. Short-term evidence suggests that LCDs provide rapid weight loss, favoring metabolic health and burning up to 300 extra calories compared to isocaloric diets with higher carbohydrate content [25,46]. Thus, the adoption of LCDs in individuals with overweight or abdominal obesity may stem from the dissemination of these potential outcomes. However, a recent meta-analysis showed that this diet makes little or no difference in weight reduction and cardiovascular risk factors in the short and long term compared to conventional ones [47]. These controversial findings may be due to the cross-sectional nature of the studies, limiting a deeper understanding of LCD benefits in different populations.

A history of smoking conferred a higher chance of following an LCD. The adoption of an LCD was more likely among individuals with a history of smoking. However, in a study by Clarke [48], comparing low-carb, high-fat diets to “other diets” and “no diet”, no significant difference was observed in smoking history between the groups. Our association can be explained by the progressive loss of taste that occurs as a direct consequence of cigarette use, contributing to a sensory preference for diets rich in saturated fats and alcohol, with lower consumption of carbohydrates and proteins [44,49,50]. In addition, there is evidence that an LCD can result in more severe symptoms of nicotine withdrawal [51].

Low-fat diets

Our study shows that individuals of non-White race/color have lower probabilities of following an LFD. Cultural differences influence dietary behaviors and patterns, thus contributing to disparities in fat intake and its food sources [52,53]. Furthermore, Indigenous, Black, and Brown individuals generally have lower incomes and less access to formal education [54,55,56], factors directly influencing overall dietary choices.

In women, being over 52 years old reduced the chance of adopting an LFD. One plausible explanation is that the pleasure of eating, as well as the freedom of choice, influences the dietary behavior of middle-aged and elderly populations [57,58]. Despite that, adopting the LFD pattern has shown benefits related to chronic diseases like breast cancer, coronary heart disease, and diabetes in middle-aged women, as evidenced by observational data from the Women’s Health Initiative cohorts [30].

More than half of the Brazilian population engages in minimal or no LTPA [35] and has a dietary pattern with high loads of food rich in fats, which may explain the lower chance of men with low LTPA following an LFD. Another explanation is that moderate and high physical activity levels and the consumption of fruits and vegetables, an indicator of a healthy diet, are accumulated by the same individuals [59], leading those with lower physical activity levels to be less concerned about overall nutrition [60].

This study presents some limitations, one of them being its cross-sectional nature, which makes it difficult to establish causality. Furthermore, although the FFQ is the most appropriate instrument in epidemiological studies, it may overestimate dietary intake. Despite the mentioned limitations, we consider the strengths of this study its use of a high-quality protocol and the rigorous training of the professionals involved in data collection. Regarding the use of the FFQ, to mitigate potential biases, rigorous quality control measures were implemented, such as the exclusion of participants with intake values considered implausible. The methods employed in this study, such as standardized data collection, established procedures for interviews, and precise anthropometric measurements, enhance its internal validity. Furthermore, training was conducted periodically at all ICs to maintain data quality.

## 5. Conclusions

This study provides evidence of the factors associated with LCDs and LFDs in the ELSA-Brasil population, a large cohort of active and retired Brazilian civil servants. In summary, considering the factors—some of them modifiable—that can lead individuals to follow restrictive diets beyond clinical and nutritional guidelines proved to be relevant, as age range, socioeconomic disparities, clinical health parameters, and levels of physical activity were associated with these choices. It is hoped that these results will promote further studies with the aim of understanding the intrinsic and extrinsic influences on the individual that impact restrictive dietary choices. In addition, we hope that our findings will help in the development of effective public health strategies, adapted to individual preferences and behaviors, aiming at the promotion of nutritional intervention in the population and the achievement of the desired outcomes by those who practice LCDs and LFDs.

## Figures and Tables

**Table 1:** Sociodemographic characteristics, health, and lifestyle habits according to low-carb diets compared to the usual and low-fat diets compared to the usual, stratified by sex (ELSA-Brasil 2024).

Variables	Total (*n* = 11,294)	Female		*p*-Value *	Male		*p*-Value *	Female		*p*-Value *	Male		*p*-Value *
		Low-Carb	Usual		Low-Carb	Usual		Low-Fat	Usual		Low-Fat	Usual	
		974 (15.7)	5202 (84.3)		984 (19.3)	4133 (80.7)		2276 (36.8)	3900 (63.2)		1946 (38.0)	3171 (62.0)	
Age (years)				**<0.001**			0.634			**<0.001**			**<0.001**
<52	6157 (54.5)	585 (60.1)	2780 (53.4)		531 (53.9)	2261 (54.7)		1415 (62.2)	1950 (50.0)		1132 (58.2)	1660 (52.3)	
≥52	5136 (45.8)	389 (39.9)	2422 (46.6)		453 (46.1)	1872 (45.3)		861 (37.8)	1950 (50.0)		814 (41.8)	1511 (47.7)	
Race/skin color				**<0.001**			**<0.001**			**<0.001**			**<0.001**
White	6162 (55.2)	618 (63.8)	2713 (52.7)		654 (67.5)	2177 (53.4)		1387 (61.4)	1944 (50.4)		1262 (65.7)	1569 (50.2)	
Non-white	5001 (44.8)	350 (36.2)	2435 (47.3)		315 (32.5)	1901 (46.6)		872 (38.6)	1913 (49.6)		658 (34.3)	1558 (49.8)	
Marital status				0.269			0.753			**<0.001**			0.999
Single	365 (13.9)	43 (14.3)	217 (13.0)		27 (17.3)	78 (15.4)		101 (14.9)	159 (12.3)		40 (15.9)	65 (15.8)	
Married	1825 (69.5)	208 (69.3)	1110 (66.7)		116 (74.4)	391 (77.3)		475 (70.2)	843 (65.5)		192 (76.5)	315 (76.6)	
Divorced	437 (16.6)	49 (16.3)	338 (20.3)		13 (8.3)	37 (7.3)		101 (14.9)	286 (22.2)		19 (7.6)	31 (7.6)	
Income strata				**<0.001**			**<0.001**			**0.002**			**<0.001**
Low	3926 (34.8)	227 (23.3)	1793 (34.5)		240 (24.4)	1666 (40.3)		686 (30.1)	1334 (34.2)		115 (47.1)	1791 (36.8)	
Intermediary	3357 (29.7)	320 (32.9)	1511 (29.1)		324 (32.9)	1202 (29.1)		720 (31.6)	1111 (28.5)		67 (27.5)	1459 (29.9)	
High	4004 (35.5)	427 (43.8)	1895 (36.4)		420 (42.7)	1262 (30.6)		870 (38.3)	1452 (37.3)		62 (25.4)	1620 (33.3)	
BMI (kg/m^2^)				**0.005**			<0.001			0.444			**<0.001**
Normal weight	4040 (35.8)	339 (34.8)	2062 (39.7)		237 (24.1)	1400 (33.9)		899 (39.5)	1502 (38.5)		558 (28.7)	1079 (34.1)	
Overweight	7249 (64.2)	634 (65.2)	3138 (60.3)		746 (75.9)	2731 (66.1)		1376 (60.5)	2396 (61.5)		1387 (71.3)	2090 (65.9)	
Waist Circumference				**0.010**			<0.001			0.187			**<0.001**
Normal	4102 (36.3)	246 (25.3)	1524 (29.3)		346 (35.2)	1984 (48.0)		675 (29.7)	1095 (28.1)		814 (41.8)	1516 (47.8)	
Elevated	7191 (63.7)	728 (74.7)	3677 (70.7)		638 (64.8)	2148 (52.0)		1601 (70.3)	2804 (71.9)		1132 (58.2)	1654 (52.2)	
Diabetes mellitus				**<0.001**			0.658			**0.001**			0.124
Yes	834 (7.4)	36 (3.7)	348 (6.7)		83 (8.4)	367 (8.9)		112 (4)	272 (7)		156 (8)	294 (9.3)	
No	10460 (92.6)	938 (96.3)	4854 (93.3)		901 (91.6)	3767 (91.1)		2164 (96)	3628 (93)		1790 (92)	2877 (90.7)	
Hypertension				**<0.001**			0.419			**<0.001**			**0.001**
Yes	3990 (35.3)	254 (26.1)	1688 (32.4)		405 (41.2)	1643 (39.7)		633 (27.8)	1309 (33.6)		720 (37)	1328 (41.9)	
No	7305 (64.7)	720 (73.9)	3514 (67.6)		579 (58.8)	2490 (60.3)		1643 (72.2)	2591 (66.4)		1226 (63)	1843 (58.1)	
Health status				**0.021**			**0.030**			**0.003**			**0.001**
Good/very good	9167 (81.2)	819 (84.1)	4190 (80.6)		825 (83.8)	3333 (80.6)		1902 (83.6)	3107 (79.7)		1627 (83.6)	2531 (79.8)	
Regular	1925 (17.1)	142 (14.6)	896 (17.2)		143 (14.5)	744 (18.1)		334 (14.7)	704 (18.1)		292 (15.0)	595 (18.8)	
Bad/very bad	199 (1.8)	13 (1.3)	115 (2.2)		16 (1.6)	55 (1.3)		40 (1.8)	88 (2.3)		27 (1.4)	44 (1.4)	
Leisure-time physical activity				0.235			**0.002**			0.250			0.023 *
Light	8442 (74.8)	743 (76.3)	4055 (78)		661 (67.2)	2981 (72.2)		1777 (78.1)	3021 (77.5)		1374 (70.6)	2268 (71.6)	
Moderate	1723 (15.3)	138 (14.2)	730 (14)		175 (17.8)	680 (16.5)		301 (13.2)	567 (14.6)		307 (15.8)	548 (17.3)	
Vigorous	1124 (9.9)	93 (9.5)	413 (8)		148 (15.0)	470 (11.3)		197 (8.7)	309 (7.9)		264 (13.6)	354 (11.2)	
Smoking				**<0.001**			**<0.001**			**<0.001**			0.224
Never smoked	6478 (57.4)	534 (54.8)	3325 (63.9)		452 (45.9)	2165 (52.4)		1360 (59.8)	2499 (64.1)		983 (50.2)	1634 (51.6)	
Former smoker	3406 (30.2)	281 (28.8)	1311 (25.2)		359 (36.5)	1455 (35.2)		606 (26.6)	986 (25.3)		682 (35.1)	1132 (35.7)	
Current smoker	1410 (12.5)	159 (16.3)	566 (10.9)		173 (17.6)	512 (12.3)		310 (13.6)	415 (10.6)		281 (14.4)	404 (12.7)	

Data expressed as n(%). * Chi-square test. Values in bold are statistically significant. BMI = body mass index.

## Data Availability

The data presented in this study are available on request from the corresponding author. The data are not publicly available due to ethical reasons.
